# Developing and evaluating a young adult peer mentor training program

**DOI:** 10.1016/j.hctj.2026.100131

**Published:** 2026-03-09

**Authors:** Beth H. Garland, Demonica L. Jones, Cassandra J. Enzler, Jean L. Raphael, Albert C. Hergenroeder, Rachel S. Wolfe, Sarah C. Graham, Constance M. Wiemann

**Affiliations:** a Department of Pediatrics, Baylor College of Medicine, One Baylor Plaza, Houston, TX 77030, United States; b Biostatistics, University of Texas M.D. Anderson Cancer Center, 7007 Bertner Avenue, Unit 1689, Houston, TX 77030, United States

**Keywords:** Mentor training, Emerging/young adults, Health care services and utilization, Health promotion and prevention, Chronic illness, Pilot/feasibility

## Abstract

Improving self-management skills for adolescents and emerging adults (AEA) with chronic health conditions is critical for successful transition to adult-based care. Peer-mentoring has been effective in improving the self-management skills of AEA. However, the components of training a successful young adult peer mentors (YAPM), especially interpersonal aspects of the role, such as communication skills and navigating mentee ambivalence are not well understood. This descriptive study created and pilot-tested a training curriculum for YAPM as part of a larger study to build self-management skills in the context of healthcare transition from pediatric to adult care. Five YAPM completed the training curriculum. The curriculum included four components: 1) organizational policies and research ethics, 2) healthcare transition content and knowledge, 3) interpersonal training for the YAPM role with a focus on using Motivational Interviewing, and 4) ongoing support. Trainings included didactic, group activities, and formal simulation. YAPM rated the training components as useful, especially interpersonal training. Skill development during simulation was measured with a standardized observational coding system. Each YAPM improved in unique ways during simulation. Simulation materials are provided to allow for further development and application in future YAPM training development. This formal mentor training curriculum has the potential to clarify expectations, provide support, and help navigate the challenging interpersonal components of the YAPM role, thereby potentially increasing the effectiveness of YAPM communication with AEA to improve self-management skills.

## Highlights

• Development and evaluation of a comprehensive, adaptable training program for YAPM.
• Novel simulation practices for interpersonal and communication skill development for YAPM.
• YAPM rated interpersonal training component as useful.
• YAPM showed individual improvements in communication skills.

## 1 Introduction

Young adults with chronic health conditions who have successfully transitioned to adult care have increasingly been employed to help adolescents and emerging adults (AEA) learn the self-management skills needed to function within the adult healthcare system. The peer mentor relationship is unique because its foundation is having similar health-related issues (“lived experience”), thus moving beyond the role of a peer educator.^1^ Shared experience and knowledge coupled with social and emotional support are frequently-cited benefits of peer mentorship, with studies identifying values such as: talking to someone ‘who has gone through what you're going through,’ receiving answers to questions and practical information, reassurance, encouragement and increased confidence, support, reducing loneliness, and hope for the future.^2^, ^3^, ^4^, ^5^, ^6^ In addition, the use of young adult peer mentors (YAPM) has led to improved AEA self-management, including increased self-efficacy to manage their illness,^1^, ^7^ greater perceived health self-management skills,^8^ and served as an accountability for further transition learning and facilitation from parent-managed to AEA-managed care.^9^

Successful training techniques to improve facilitation and efficacy of peer mentors for adults with HIV^10^ and youth with emotional/behavioral difficulties^11^ have been reported, but details of how best to train YAPM to be successful peer mentors to AEA with chronic medical conditions are lacking. In the two studies presented above, the authors indicated the number of hours of training and the broad themes of training (e.g., communication skills) with limited details to allow for replication. Peer mentor programs for AEA with chronic conditions need consistent evaluation to gain consensus on best training practices to improve efficacy^12^ and protect and support both the mentee and mentor.

The YAPM role is complex and being formally defined, leading to a wide array of skills that a YAPM may need in their specific job. Historically, YAPM training has focused on the content/knowledge to be shared (e.g., self-management skills, how to navigate transition to adult-based care). More recent YAPM studies have focused on training in communication strategies *for how and when* to share the content/knowledge (e.g., active listening).^13^ YAPM report a desire for more information and training to help them navigate mental health support with mentees, relationship boundaries, use of personal disclosure, different mentee personalities, and termination/closure of the mentor relationship.^5^, ^7^, ^14^, ^15^ Klodnick and colleagues^13^ describe challenging aspects of the relationships experienced in the YAPM role, such as how to share lived experience (identifying the reason for sharing, sharing while navigating personal emotions about the experience), balance listening with sharing, and establish and maintain boundaries with the AEA. Given that interpersonal connections are at the core of the YAPM role, interpersonal difficulties may arise in the role. YAPM are seeking extensions of their training to include interpersonal skills training. A crucial next step is to begin to define this training more formally in YAPM programs.

When training a YAPM in the communication skills needed to support potential behavioral changes in AEA (e.g., behaviors related to self-management and transition to adult-care), a key consideration is the role of mentee ambivalence. AEA ambivalence or uncertainty toward self-management could impact the acceptance and implementation of knowledge provided by the YAPM. Motivational Interviewing (MI) is one health behavior change tool that a YAPM could use to address ambivalence while also empowering an AEA in health-related changes. MI is designed as a guiding communication tool to discuss and empower recipients toward behavior change in a partnering, accepting, compassionate, and empowering environment utilizing the recipient’s own values and motivation to support and reinforce change talk toward adaptive change.^16^ Successful MI for AEA with chronic conditions has resulted in increased adherence to medical regimen, reduced symptom levels, improved quality of life, and positive well-being.^17^, ^18^, ^19^, ^20^ To date, several programs have reported teaching MI skills to peer mentors.^21^, ^22^, ^23^, ^24^ Various MI skills were utilized by YAPM across these studies; a variety of teaching modalities were also explored including lecture, roleplay, coaching, video viewing, and skill testing. However, there remain gaps in our understanding of how to best prepare YAPM to use MI to communicate with AEA and establish appropriate relationships in YAPM programs aimed at improving healthcare transition skills.

To address these training gaps, the authors created a multi-modal training curriculum for YAPM working with AEA with chronic conditions as part of a larger group-based intervention to increase self-management skills for AEA.^25^, ^26^ The entire training is provided to the reader, with specific emphasis and detail on the interpersonal training component. Unique to this training is the incorporation of simulation practice in formal interpersonal skills training. The purpose of this study was to describe the creation of the formalized YAPM training curriculum and its evaluation, including YAPM satisfaction and an assessment of changes in YAPM skills.

## 2 Methods

### 2.1 Participants

Five YAPM were hired to implement a group-based mentor curriculum to AEA ages 17–22 years with renal, rheumatologic, or inflammatory bowel disease. The group intervention included four, interactive, 4-hour sessions over 4–6 weeks, where YAPM collectively co-facilitated group activities, discussions, and didactics with a cohort of mentees. YAPM also completed individual check-ins with assigned mentees between group sessions.^25^ YAPM were recruited either by personal recommendation by health care providers or in response to an advertised position for a community health worker. The YAPM position required lived experience with a chronic condition, defined as either personal experience and diagnosis or lived experience gained from a friend or family member with a chronic condition in one of the three services receiving the intervention. YAPM candidates completed a written application and interview as part of the hiring process. Of the five YAPM hired, four were female. Four had direct lived experience with a chronic condition, the remaining YAPM had lived experience via a close peer with a chronic condition. YAPM were 22–23 years of age. Protocols for evaluating the training program were approved by the Institutional Review Board for Human Subjects Research, and all YAPM provided written, informed consent.

### 2.2 Procedure

This section describes the development of the training curriculum, the training curriculum itself, and the methods used to evaluate it.

#### 2.2.1 Curriculum development

Psychology, medicine, public health faculty and research coordinators participated in curriculum development. Literature review identified mentor training programs for peer mentors in healthcare and mental healthcare, including those used with adults.^27^, ^28^, ^29^, ^30^, ^31^ Two investigators attended a national mentor training conference aimed at identifying critical components of peer mentor and youth development programs. The interdisciplinary team met weekly for nine months to build training content. The final YAPM training program included didactic instruction with simulation sessions to practice interpersonal skills.

#### 2.2.2 Training curriculum components

The training curriculum included: 1) organizational policies and research ethics, 2) healthcare transition content and knowledge, 3) interpersonal training for the YAPM role, and 4) ongoing support. (see Table 1).

**Table 1:** YAPM training components and materials.

Organizational policies and research ethics • New Employee Online Trainings (organizational specific and required) a. Confidentiality Orientation b. Employee Relations Orientation c. Security Orientation d. Privacy and Security (HIPAA) training e. Review and sign Social Media Policy • Collaborative Institutional Training Initiative (CITI^32^, ^33^, ^34^) Online Trainings a. Human Subjects Research for Research Investigators b. Health Information Privacy and Security- Biomedical c. Responsible Conduct of Research for Students and Fellows Healthcare transition content and knowledge • Project Introduction Meeting • Independent Reading a. Review research team’s publications b. Review *Grant Project Narrative* c. Review *Group Intervention Curriculum Manual* d. Review educational materials and web resources for each diagnosis e. Review GotTransition.org, especially for transition components and 6 core elements^35^ f. Review six In-Depth Interview Transcripts from intervention development across three medical specialties • Attend AEA/Caregiver Advisory Board Meeting Interpersonal training • MentoringCentral.net Online Trainings^36^ a. Building the Foundation b. Ethics & Safety • Motivational Interviewing training a. 2 pre-training simulations (phone, face-to-face) b. Single MI didactic and practice c. 2 post-training simulations (phone and face-to-face) • Group Facilitation Training (single session) Ongoing Support • 3-month workshop refresher on training components above • Weekly, group supervision with integrated problem solving

##### 2.2.2.1 Organizational policies and research ethics

YAPMs participated in self-paced learning on organizational policies and research ethics training, including presentations on confidentiality, employee relations, HIPAA privacy and security, and the use of social media. In addition, YAPM completed modules of the Collaborative Institutional Training Initiative (CITI) program.^32–34^The CITI modules are a federal compliance requirement given that YAPM were managing human subjects data. In addition, as the YAPM were new to research, this training was to sensitize them to the importance of maintaining confidentiality, and to further contribute to their development as professionals.

##### 2.2.2.2 Healthcare transition content and knowledge

YAPM completed independent readings on 1) healthcare transition, with specific focus on concepts in transition and the six core elements^35^; 2) common disease management skills needed for the conditions being targeted in the larger intervention (e.g., medication management, making and keeping clinic appointments, identifying an adult provider, understanding warning signs of emergent situation with their diagnosis); 3) recent empirical literature; and 4) the grant narrative for the broader intervention project. YAPM also read six caregiver and AEA transcribed interviews that had been conducted as part of the process of developing the intervention.^26^ These interview transcripts offered insights into the challenges of self-management for each condition. YAPM attended one AEA or caregiver advisory board meeting to reinforce the concepts important to the population of the intended intervention. Weekly meetings between YAPM and the curriculum development team provided opportunities for discussion of what the YAPM were reading and learning.

YAPM also reviewed the draft of the manual for the group-based intervention they were being trained to deliver. The manual included goals and instructions for implementing each session with examples of language and potential activities. YAPM discussed each session of the group intervention curriculum and practiced with roleplay. During these discussions, YAPM were encouraged to improve or create intervention activities to utilize their expertise from both a generational and lived experience perspective.

##### 2.2.2.3 Interpersonal training

This training included a focus on the mentor role, mentor expectations, potential pitfalls, and communication styles that foster engagement and promote motivation for behavioral change for individual and group-based interventions. The interpersonal training included the following three components: Mentoring Central online modules, workshops, and simulation.

YAPM completed two modules of the Mentoring Central^36^ online trainings that focused on goals of mentoring and ethics of mentor relationships. This training was not geared toward healthcare mentors, but rather globally to the role of being a mentor to an adolescent. A group discussion followed and provided strategies for how to respond to personal questions asked of YAPM by mentees, how to navigate personal disclosure, and how to maintain appropriate and safe boundaries with respect to the unique nature of this mentor relationship.

Two workshops were developed to focus on 1) MI strategies and 2) group facilitation strategies. The MI training taught, modeled, and allowed for practice with verbal strategies to facilitate positive mentee behavioral change. The session objectives included understanding the process of change, verbal skills (e.g., open questions, reflections, affirmation, emphasizing autonomy), recognizing and responding to change talk, limiting advice-giving/increasing use of permission seeking, and alternatives to personal disclosure. Group facilitation training included didactic instruction and skill modeling by the facilitator. The group facilitation training sessions were divided into four content sessions: logistics (room layout, positioning of the YAPM), strategies for engagement that reinforced the MI content (reflective listening, summarizing, validation, open-ended questions), group management challenges (setting ground rules, responding to challenging behaviors), and YAPM issues (competing ideas between YAPM, conflict resolution between YAPM).

To practice MI skills, YAPM participated in four simulations with trained standardized mentee actors. Simulations were created for two in-person interactions and two phone call check-ins, which were aligned with the broader intervention program, as YAPM were required to complete phone calls with their mentee between group sessions as well as work individually with mentees during structured activities in the group curriculum. Each YAPM completed two simulations (one phone, one in-person) before the MI training and then completed two post-MI training simulations (one phone, one in-person) with different standardized mentee actors. Each YAPM participated individually in these simulations, as opposed to completing a group simulation, which maximized each YAPM’s practice of skills and prevented differences in YAPM dominance or personality from overshadowing another YAPM’s opportunity to practice. See Appendix A for all training materials and detailed protocol of the simulations.

Following each simulation, YAPM and standardized mentee actors completed written reflections on the encounter. Study staff and the standardized mentee actor offered feedback to the YAPM during debrief sessions immediately following simulations. These 15–20 min, in-person debrief sessions included discussion with YAPM on strengths and areas for improvement. All mentee simulations were video- or audiotaped, transcribed, and reviewed by study staff. Over 1–2 weeks after the simulation, YAPM watched/listened to their recordings independently and identified MI-congruent behaviors. These codes and observed skills were discussed in one-on-one, in-person supervision meetings (1 h, per YAPM) to continue to reinforce MI skills.

##### 2.2.2.4 Ongoing support

To maintain intervention fidelity, YAPM completed a refresher training approximately three months later. The refresher training included a workshop and practice roleplay. During their time as a mentor, YAPM also participated in ongoing, weekly group supervision to discuss challenges encountered, problem solve barriers, review and plan curriculum, as well as celebrate successes.

In summary, the YAPM completed the organization/ethics, transition knowledge, and interpersonal trainings, including simulations, within the first month of hire. A refresher training was provided three months after hire, once YAPM were implementing the intervention. YAPM employment length varied. The shortest length of hire was 10 months; the longest length of hire was three years. YAPM completed an exit interview at the end of their hire.

#### 2.2.3 Evaluation of the training curriculum

##### 2.2.3.1 Interpersonal training component satisfaction and comfort in role

YAPM rated the utility of each of the training components using 4-point Likert scales from “not at all useful” to “very useful.” No neutral/middle option was provided. YAPM reported their rating of comfort in the role of the mentor before and after the training using a single item 4-point Likert scale ranging from “not at all comfortable” to “very comfortable.” Two questions during exit interviews specifically asked what was most helpful about the training received and what could have been more helpful.

##### 2.2.3.2 Effectiveness of interpersonal training

The interpersonal training was evaluated with two behavioral observations (behavioral counts and amount of time the YAPM spoke in the encounter) and with one written evaluation by the standardized mentee actor.

###### 2.2.3.2.1 Behavioral counts

Simulation experiences were evaluated for skill development. Simulations were coded using behavioral count concepts defined by the Motivational Interview Treatment Integrity (MITI) coding system (4.2.1).^37^ One adaptation was condensing complex and simple reflections into one count due to skill level of the coders and YAPM. Several behaviors were targeted for desirable improvement over the course of training: Reflections, Affirmations, Emphasizing Autonomy, and Seeking Collaboration. Several behaviors were targeted as undesirable and hypothesized to decrease from pre- to post-training, for example, Persuade and Confrontation. MITI codes of Giving Information and Questions were counted, and during training these two skills were emphasized to be used with purpose/intention and may reduce in frequency in favor of other MI skills (e.g., reflections and affirmations). Open-ended questions were encouraged and modeled; however, these were not coded separately as there is no distinction between open- and closed-ended questions on the MITI. Table 2 provides a basic description of each behavior, especially as it relates to the role of the YAPM. However, these are not to replace the operational definitions that are described by Moyers and colleagues^37^ but rather to give context in the manuscript to the results of this study.

**Table 2:** Descriptions of MI behavioral skills evaluated during simulation.

**Behavior**	**Definition as it applies to the YAPM role**
Behaviors hypothesized to increase	
Reflection	YAPM providing reflective listening statements based on mentee's statement. Could be either a complex or simple reflection as defined by the MITI
Affirmation	YAPM emphasizing a strength or effort of the mentee
Emphasizing Autonomy	YAPM honoring mentee’s freedom of choice and control in decision-making. Often used less frequently. Hypothesized to increase in frequency to a lesser degree than reflections and affirmations.
Seeking Collaboration	YAPM acknowledging mentee expertise and seeking permission to share information. Often used less frequently. Hypothesized to increase in frequency to a lesser degree than reflections and affirmations.

Behaviors hypothesized to decrease	
Persuade	YAPM attempting to change mentee’s opinion or YAPM self-disclosure with intention for mentee to do the same behavior
Confrontation	YAPM statements of blame, criticism, arguing with mentee

Behaviors that are hypothesized to change as YAPM demonstrate other behaviors	
Giving Information	YAPM giving feedback or information in general terms, not directed to the mentee specifically or a personal self-disclosure by the YAPM
Questions	YAPM utterances that end with intonation up and promote the mentee to offer an answer related to the prompt Open and Closed Questions were included in this count. Reflections that end with intonation up are counted in this category.

Simulations were transcribed and checked for accuracy. Three raters, not YAPM, were trained as coders on practice scripts utilized from a separate research project conducted by the authors. After coding training and practice, each simulation transcript was coded independently by two raters who were blinded to pre-or post-training status of the mentor in the video. Behavioral count codes were compared between raters, and discrepancies were resolved by discussion, review of video footage, or utilization of the third rater.

###### 2.2.3.2.2 Talk time

In addition to MITI codes, total mentor talk time was recorded for the duration of each simulation. Talk time was operationalized as an informal measurement of a guiding style of communication, with a decrease in mentor talk time suggestive of an increase in mentee engagement to share or reflect. Talk time was a running time of the seconds the mentor spoke during the full encounter. To account for the varying lengths of encounters, percentage talk time was defined as the number of seconds the mentor was recorded talking divided by the total number of seconds in the encounter. If the mentor uttered two words or more, this was captured as part of talk time. Single word utterances (e.g., “okay”) were not recorded in the time.

###### 2.2.3.2.3 Standardized mentee actor reflection

The standardized mentees participating during simulations provided feedback on mentor performance in broad interpersonal domains, such as comfort in talking with the YAPM, ratio of perceived change talk, and partnership. Likert responses were on a 6-point scale ranged from “strongly disagree” to “strongly agree.” Ratings were collapsed across vignettes and compared between pre- and post-training. See Appendix A for specific standardized mentee rating forms.

### 2.3 Analyses

Descriptive statistics were the primary data analysis. Representative quotes extracted from exit interview transcripts were identified.

## 3 Results

### 3.1 Interpersonal training component satisfaction and comfort in role

The training components were generally rated as very useful. (see Table 3). Perceived comfort with the mentor role at pre-training was rated as slightly/somewhat comfortable (60%) and moderately/mostly comfortable (40%). Post-training, each mentor increased their rating by at least one Likert level, with 40% of YAPM reporting moderately/mostly comfortable, 40% of YAPM reporting very comfortable, and one YAPM who indicated both mostly and very comfortable (selected two options). Table 4 presents comments about the helpfulness of the training reported by YAPM during exit interviews following their time as a mentor.

**Table 3:** YAPM ratings of the utility of training components.

	Not at all useful	Slightly useful	Moderately useful	Very useful
Mentor Central Training	0%	40%	20%	40%
Motivational Interviewing	0%	0%	0%	100%
Group Facilitation	0%	20%	0%	80%
Simulation Practice	0%	0%	0%	100%

**Table 4:** Exit interview quotes regarding training helpfulness.

**Question**	
What was most helpful about the training?	
	• I think what helped me personally the most was the standardized [mentee] stuff. I think being able to hear myself, see myself, and be in those moments even though they were a little more artificial than what we actually encountered, that was so helpful. It didn’t necessarily build my confidence, but looking back I can see now like, my growth. • I think [training] prepared me in being able to give better advice and guide someone to better themselves without actually pushing them and making it seem like I'm controlling them. • When we first started, we had all the training process that we had to go through, and I think that really helped me in my role as a mentor. All of the feedback was helpful and knowing what to say to them, what not to say.

What could have been more helpful to training?	
	• I do feel that there should’ve been some kind of training on how much or what we as mentors could share about our stories. • I think we could have had like more realistic interactions. 'Cause some of them were like the 'perfect patients'. • I think it’s just a public speaking fear that I have. I don’t know. I guess I would have liked more of [that] training, I think. It would have been helpful. • Maybe two or three more [mentee simulations] would be helpful as well because [the facilitator’s simulation example video] was helpful as well, very helpful. I feel like if I had more of those, it would have boost up my confidence a little more.

### 3.2 MI skill demonstration and improvement

Table 5 contains the behavior count frequencies of MITI codes by YAPM. Behavioral counts are presented separately for each YAPM for several reasons: 1) this pilot has a small sample of YAPM, and 2) each YAPM likely represents a unique, typical YAPM who may have strengths/areas of improvements. With respect to overall patterns, reflections were the only skill to increase between pre- and post-assessment for all YAPMs. All YAPM also improved in at least two desireable behaviors. Affirmations, seeking collaboration, and emphasizing autonomy increased at post-training for two, three, and two YAPM, respectively. The Persuade code decreased by post-training for four YAPM. The use of questions and giving information, which were predicted to decrease based on the use of other skills did so for all YAPM and two YAPM, respectively. Confrontation was not observed in any YAPM pre- or post-training.

**Table 5:** MI skill changes between baseline and post-MI training.

	**Frequency** Baseline*	**Frequency** **Post-MI training**
**Mentor 1**		
Desirable interpersonal skills		
Reflections	**6**	8**
Affirmations	6	2
Seek Collaboration	**2**	**4**
Emphasize Autonomy	1	0

Undesired interpersonal behaviors***		
Persuade	**8**	**4**
YAPM % talk time	**46.2%**	**43.1%**

Skills to use less frequently in favor of Reflections and Affirmations		
Questions	63	51
Giving Information	0	0

Mentor 1’s noted strengths included being inquisitive, often with questions. They noted feeling hesitant to give advice and did not use self-disclosure; however, they did work toward problem solving. They worked to decrease the use of persuasion and increase seeking collaboration statements. Supervision focused on strategies for giving information, crafting genuine reflections, and increasing reflections.		

	**Frequency** Baseline*	**Frequency** **Post-MI training**
**Mentor 2**		
Desirable Interpersonal Skills		
Reflections	**3**	**5**
Affirmations	**0**	**3**
Seek Collaboration	1	0
Emphasize Autonomy	0	0

Undesired interpersonal behaviors		
Persuade	**7**	**4**
YAPM % talk time	**45.9%**	**44.5%**

Skills to use less frequently in favor of Reflections and Affirmations		
Questions	48	46
Giving Information	3	1

Mentor 2 demonstrated minimal changes between pre and post training. They actively worked toward increasing reflections and affirmations, and decreasing statements of persuasion. General supervision also included increasing warmth and engagement with mentees through open-ended questions.		

	**Frequency** Baseline*	**Frequency** **Post-MI training**
**Mentor 3**		
Desirable Interpersonal Skills		
Reflections	**4**	**6**
Affirmations	**2**	**7**
Seek Collaboration	**0**	**2**
Emphasize Autonomy	**0**	**1**

Undesired interpersonal behaviors		
Persuade	14	15
YAPM % talk time	54.4%	54.9%

Skills to use less frequently in favor of Reflections and Affirmations		
Questions	44	31
Giving Information	1	3

Mentor 3 expressed high comfort with sharing about their condition and often included statements of self-disclosure, often in the form of persuasive statements that a mentee could do to assist with a particular concern. Ongoing supervision included increasing reflections and crafting genuine affirmations as well as formulating strategies for giving information utilizing permission seeking and involving the mentee in problem solving.		

	**Frequency** Baseline*	**Frequency** **Post-MI training**
**Mentor 4**		
Desirable Interpersonal Skills		
Reflections	**24**	**25**
Affirmations	3	0
Seek Collaboration	**1**	**3**
Emphasize Autonomy	2	1

Undesired interpersonal behaviors		
Persuade	**11**	**6**
YAPM % talk time	**52.9%**	**37.0%**

Skills to use less frequently in favor of Reflections and Affirmations		
Questions	47	36
Giving Information	2	1

Mentor 4 had prior involvement with Motivational Interviewing concepts and ease with reflections that came from their perception of the value of reflections in conversations. They also experienced a comfort with sharing about their experiences, often leading to persuasion for ideas about how to fix a current difficulty; however, integrated more open ended questions to evoke ideas from the patient at post-assessment. Ongoing supervision focused on the direction of reflections toward change and strategies for providing information or problem solving with a patient (rather than for a patient).		

	**Frequency** Baseline*	**Frequency** **Post-MI training**
**Mentor 5**		
Desirable Interpersonal Skills		
Reflections	**7**	**9**
Affirmations	1	0
Seek Collaboration	1	0
Emphasize Autonomy	**0**	**1**

Undesired interpersonal behaviors		
Persuade	**26**	**19**
YAPM % talk time	**72.5%**	**66.6%**

Skills to use less frequently in favor of Reflections and Affirmations		
Questions	64	30
Giving Information	1	17

Mentor 5 was most often oriented to problem solving and self-disclosure of lived experience. They were inquisitive to the mentee’s experience, originally through closed ended questions, and actively worked to include more open-ended questions of exploration with the mentee. Ongoing supervision included decreasing persuasive phrases toward more inclusion of mentee in the problem-solving responsibility. In addition, broader topics of leaving a conversation with no selected solution by the mentee (e.g., supportive space in decision making) and the value of reflections to evoke additional information were foci in supervision.		

* In-person and phone simulation data combined

** Bolded values indicate change in the desired direction

*** Confrontation behavioral counts are not listed in the table because these were not an observed skill by any YAPM.

While YAPM had a maximum of 20 min in each simulation, some exited the room early if they felt they accomplished the tasks of the simulation before time expired. For in-person simulations pre- and post-training, encounters ranged between 8 and 20 min. For phone simulations pre- and post-training, encounters ranged from 5 to 12 min. YAPM reported that the phone calls were more difficult to cultivate behavior change, and these encounters were noted to have shorter engagement times. YAPM talk time decreased from pre- to post-training for four of the five YAPM. (see Table 5).

Standardized mentee ratings of adaptive skills and attitudes exhibited by the YAPM increased from pre- to post-training in four of the five mentors. The largest increase for any mentor on a single items was 2.5 likert points (Mentor 5) (See Table 6).

**Table 6:** Average standardized mentee ratings of YAPM performance by vignette and time.

**Mentor 1**			
**Observation**	Baseline*	**Post-MI training**	**Likert Change**
This mentor took time to understand my side/position and personal reasons for my actions.	4.5	4.5	0.0
This mentor allowed me to think of my own solutions to my problem before giving me his or her opinion or advice.	3.5	4.5	1.0
I found myself talking more about change as opposed to barriers or problems with changing.	5.0	4.0	-1.0
This mentor took my opinion seriously.	5.5	5.0	-0.5
I felt comfortable expressing myself with this mentor.	4.5	4.5	0
**Global Average across all ratings**	**4.7**	**4.5**	**-0.2**

**Mentor 2**			
This mentor took time to understand my side/position and personal reasons for my actions.	3.5	4	0.5
This mentor allowed me to think of my own solutions to my problem before giving me his or her opinion or advice.	2.0	3.5	1.5
I found myself talking more about change as opposed to barriers or problems with changing.	3.0	4.5	1.5
This mentor took my opinion seriously.	5.0	4.5	-0.5
I felt comfortable expressing myself with this mentor.	4.0	4.5	0.5
**Global Average across all ratings**	**3.5**	**4.2**	**0.7**
	
**Mentor 3**			
This mentor took time to understand my side/position and personal reasons for my actions.	5.0	6.0	1.0
This mentor allowed me to think of my own solutions to my problem before giving me his or her opinion or advice.	4.0	5.5	1.5
I found myself talking more about change as opposed to barriers or problems with changing.	4.0	4.5	0.5
This mentor took my opinion seriously.	5.0	6.0	1.0
I felt comfortable expressing myself with this mentor.	5.5	5.0	-0.5
**Global Average across all ratings**	**4.7**	**5.4**	**0.7**

**Mentor 4**			
This mentor took time to understand my side/position and personal reasons for my actions.	5.5	6.0	0.5
This mentor allowed me to think of my own solutions to my problem before giving me his or her opinion or advice.	6.0	6.0	0.0
I found myself talking more about change as opposed to barriers or problems with changing.	5.5	6.0**	0.5
This mentor took my opinion seriously.	6.0	6.0	0.0
I felt comfortable expressing myself with this mentor.	6.0	6.0**	0.0
**Global Average across all ratings**	**5.8**	**6.0**	**0.2**

**Mentor 5**			
This mentor took time to understand my side/position and personal reasons for my actions.	5.0	6.0	1.0
This mentor allowed me to think of my own solutions to my problem before giving me his or her opinion or advice.	3.5	3.5	0.0
I found myself talking more about change as opposed to barriers or problems with changing.	2.0	4.5	2.5
This mentor took my opinion seriously.	5.5	6.0	0.5
I felt comfortable expressing myself with this mentor.	4.5	6.0	1.5
**Global Average across all ratings**	**4.3**	**5.2**	**0.9**

* Scores are the average of 2 likert items (telephone and in-person vignettes) on a 6-point scale. Scores could range from 1 to 6; higher scores indicate stronger agreement of the statement by the standardized mentee

** Mentor had one set of missing data for two items for one vignette. Values in these boxes indicate standardized mentee ratings for telephone post-training vignette only.

## 4 Discussion

A multi-modal training curriculum for YAPM working with AEA with chronic health conditions as they prepare to transition into adult healthcare was designed and successfully implemented with a pilot group of five YAPM.

YAPM described training components as useful, with simulation experiences and the interpersonal training receiving the highest ratings. The interpersonal training targets an essential set of skills for developing an effective YAPM. These skills, separate from the content of their expertise as a person with lived experience, help to define and give structure to the YAPM role, differentiating it from both a friendship and a healthcare provider. As a testament to the value of the interpersonal skills, YAPM recommended adding simulations with more challenging scenarios, as well as expanded training in self-disclosure and responding to personal questions from AEAs.

While there can be benefits to disclosure and sharing of experience, a delicate balance exists between when, how, and what the YAPM shares with the AEA. Each act of disclosure has the potential to impact the trajectory of an AEA’s decision-making process and autonomy growth as well as the relationship between mentee and mentor (e.g., perceived warmth, closeness, support). The YAPM’s desire for additional training on personal disclosure emphasizes the importance of providing structure and guidelines early in the role, as well as continued support by well-trained professionals. This is congruent with a broader qualitative analysis of different relationships in the YAPM role, specifically the skill building of balancing listening to sharing/self-disclosure, establishing boundaries, and managing personal emotions.^13^ Given that YAPM relationships are often utilized and valued by AEAs as emotionally supportive and accepting experiences,^3^, ^5^ these data highlight the importance of specific training in boundaries, self-disclosure, and interpersonal interaction beyond content area expertise to preserve the value of mentorship in a safe way. These interpersonal topics, which the training described in this paper provided, reinforce the importance of separating the YAPM position from that of peer/friend while supporting the expertise of the YAPM that is distinct and valuable from that of other health professionals.

With respect to skill development, all YAPM showed improvement in at least two behavioral skills (e.g., decreasing an undesirable behavior or increasing a desirable strategy) which were also observed by the standardized mentee from pre- to post-training. In addition, time the YAPM spent talking decreased, which may represent an increase in overall evoking skills to elicit information from their standardized mentee, a fundamental component of MI, as opposed to primarily inform. However, changes in specific behaviors (e.g., affirmations, emphasizing autonomy, seeking collaboration) varied with each YAPM. This suggests a unique learning trajectory of each YAPM and underscores the value of ongoing support and supervision to create specific interpersonal goals based on a YAPM’s strengths and areas in need of improvement. Ongoing supervision and booster sessions identified for unique training needs of YAPM have been reported.^13^, ^21^

### 4.1 Lessons learned

1) Ongoing training and personalized supervision was crucial to mentor improvements and comfort in their mentoring role. This is congruent with studies that have used a just-in-time asynchronous training sessions^22^ or booster sessions^21^ to support skills, ongoing supervision,^13^, ^21^ and the use of group supervision with multiple YAPM to debrief and collaborate together on ways to address tough aspects of the role.^5^, ^13^ Based on the findings, a feasible model for booster sessions could follow a 3–6–12 month cadence post-intervention, with each session focusing on reinforcement of key skills, troubleshooting barriers, and peer support. Ongoing support might also include optional quarterly check-ins or digital touchpoints to sustain engagement and retention of learned strategies. Future studies could benefit from continually monitoring YAPM skills following training and during intervention implementation to best guide the necessary frequency and intensity of booster sessions and supervisions. For projects with a large number of YAPM, it might be advisable that YAPM have one-on-one time with a supervisor or the opportunity to submit anonymous questions during trainings to support knowledge growth, especially in the context of a YAPM who may feel uncomfortable with asking a question during training. 2) There is benefit for the YAPM in understanding the purpose of each training component. A formal introduction and rationale of each component would potentially increase the relevance and acceptability of some components. 3) Continued practice with skills and curriculum delivery (e.g., public speaking practice) is indicated. Even though role-playing exercises and simulation were anxiety-provoking for some YAPM, the opportunity to practice skills and speak in front of others was highly valued. 4) Job coaching is needed and formal supervision was often mixed with a discussion, per the YAPM’s direction, on their role as an employee given that the YAPM role was often their entry into paid employment. This was an unexpected supervision topic. Examples included frustration management when working on a team, team collaboration skills, arriving on time/ahead of a meeting, coming prepared, taking appropriate notes, managing personal stressors and their impact on job performance, and appropriate dress. These topics are similar to the Koldnick and colleague’s^13^ descriptions of the YAPM role with non-peer colleagues and navigating aspects of professionalism, advocacy for the role, and learning to partner with other colleagues on a team.

### 4.2 Strengths and limitations

To the authors’ knowledge, this is the first published study to describe the development and evaluation of a training program for YAPM preparing to work with AEA with chronic health conditions. The comprehensive approach to the development of peer mentor training that included scientific literature review and collaboration of a multi-disciplinary team adds to the rigor of the curriculum development. An additional strength is the use of a training curriculum that can be modified to train YAPM working with different chronic conditions, increasing its relevance across multiple pediatric conditions. The collection of observational data using a highly validated and long-standing coding system and standardized mentee observations to evaluate skill change offers a specific and formalized viewpoint of YAPM skills and improvement potential. With respect to limitations, the mentor training curriculum described here should be further evaluated using larger, more diverse groups of YAPM. Further evaluation of skills, for example, recording and coding YAPM interactions during an intervention, would help demonstrate the potential for lasting impacts of training, further opportunity for skill refreshment, and serve as ongoing supervision content. There is a possibility of ceiling effects in mentor comfort scores, particularly given the relatively high baseline ratings observed. This may have limited the ability to detect improvements post-intervention. In future iterations, the comfort scale may need refinement to allow for greater differentiation among experienced mentors and to explore alternative or supplemental qualitative measures that capture nuance in mentor development over time. While shorter simulations may suggest increased skill or efficiency in navigating the scenario, they could also reflect disengagement or truncated participation. In our analysis, we reviewed facilitation notes and debrief summaries to better interpret simulation length. Future studies will include more robust process measures, such as fidelity checklists or time-on-task coding to better discern whether brevity reflects proficiency or other factors. Finally, the MITI, most commonly used with practitioners, may not fully capture the gradual improvement or skill development in YAPM. In addition, percent talk time may need to be evaluated in conjunction with content coding of the YAPM discussion. Given that a YAPM’s role is to share experiences and lived expertise and research suggest that mentees appreciate that sharing from their YAPM, an increase in talk time may be appropriate when done in an MI-congruent way to discuss behavioral changes. Last, this project focused on individual skill change for YAPM to promote strong individual skill; it did not evaluate for YAPM group dynamics when working together as co-leaders for a group intervention. Future research evaluating peer mentor groups could include individual and group-observed changes, given that YAPM skills may be slightly different when working in tandem with other YAPM. Overall, these pilot data contribute a comprehensive training curriculum, method of training, and important considerations for the YAPM role.

## CRediT authorship contribution statement

**Wolfe Rachel S:** Writing – review & editing, Writing – original draft, Conceptualization. **Albert C. Hergenroeder:** Writing – review & editing, Funding acquisition, Conceptualization. **Jean L. Raphael:** Writing – review & editing, Conceptualization. **Enzler Cassandra J:** Writing – review & editing, Formal analysis, Data curation. **Jones Demonica L:** Writing – review & editing, Writing – original draft, Formal analysis, Data curation, Conceptualization. **Garland Beth Hackethorn:** Writing – review & editing, Writing – original draft, Methodology, Formal analysis, Data curation, Conceptualization. **Wiemann Constance M:** Writing – review & editing, Writing – original draft, Funding acquisition, Formal analysis, Data curation, Conceptualization. **Sarah C. Graham:** Writing – review & editing, Writing – original draft, Data curation, Conceptualization.

## Consent to participate

All participants provided written, informed consent.

## Ethics approval

All procedures performed in studies involving human participants were in accordance with the ethical standards of the institutional and/or national research committee and with the 1964 Helsinki Declaration and its later amendments or comparable ethical standards. The study was approved by the Institutional Review Board for Human Subjects Research (Baylor College of Medicine; H-41767).

## Funding statement for the manuscript

This work was supported by the Health Resources and Services Administration (HRSA) of the U.S. Department of Health and Human Services (# R40MC30764–01, CMW), Project IMPAACT: Innovative Mentor Program for Achieving Autonomy and Competence in Transition. The information or content and conclusions are those of the author and should not be construed as the official position or policy of, nor should any endorsements be inferred by HRSA, HHS or the U.S. Government.

## Declaration of Competing Interest

The authors declare the following financial interests/personal relationships which may be considered as potential competing interests:

## Data Availability

The data that has been used is confidential.
