# Supplementary material for: Developing and evaluating a young adult peer mentor training program
Source: Health Care Transit. 2026 Mar 9;4:100131. doi: 10.1016/j.hctj.2026.100131 (PMC12993229; doi:10.1016/j.hctj.2026.100131)
Supplement: Supplementary file 1 — Supplementary material [file mmc1.docx]

**Appendix A: Mentor Training Simulation Materials and Methods**

This appendix provides specific details of simulation descriptions, protocol for simulation training, and the training materials.

Description of Vignettes Used for Simulations

There were four simulations as part of the YAPM training. The four simulations were standardized and semi-scripted vignettes representing potential presenting difficulties that a YAPM may face with a mentee. As noted in the methods, this peer mentor experience was in the context of a larger group intervention focused on transition. Some vignettes below may present the start of a conversation in the context of having completed a group activity of the larger intervention; however, the scenarios are generalizable to similar situations that may occur with YAPM during the process of transition to adult-baesd care and learning self-management. The table below provides the type of simulation, brief summary of presenting concern as provided to the YAPM and the Standardized mentee actor.

| **Scenario** | **Case synopsis** |
| --- | --- |
| *A: Ambivalence in goal setting*  Baseline  Face-to-face conversation | A mentee is observed to be frustrated while working on a group activity (related to goal setting), in the context of transitioning to adult-based care. After the group is complete, the mentor approaches the mentee to discuss what might be tough or the source of frustration. The mentee reveals that they are not sure where to start in thinking about their next steps for transition. |
| *B: Ambivalence toward group*  Baseline  Phone conversation | This is a scheduled check-in phone call after the first group session. The mentor had observed the mentee to seem “checked out” and possibly struggling to find value in the group meetings. |
| *C: Difficulty with describing diagnosis*  Post-training  Face-to-face conversation | The most recent group session discussed how someone might explain their diagnosis to someone new. The mentee and mentor meet to discuss this content together, specific to the mentee. The mentee discloses that sharing their diagnosis has not gone well in the past, but that they can see the value/benefit in letting others around them know. |
| *D: Difficulty with locating adult provider*  Post-training  Phone conversation | This is a scheduled check-in phone call after the second group session, where content covered selecting a new adult health care provider. The check in is specific to what the mentee has accomplished in this process and where they may feel stuck. The mentee discloses that they haven’t started the process yet. |

Simulation Protocol

*Standardized Mentee Recruitment and Training.* Standardized mentee actors were recruited from recommendations of healthcare providers in relevant subspecialities for the larger intervention and project-specific youth advisory boards. Each actor participated in a 2-hour training session to prepare for the role and one practice round with the lead author. Standardized mentees were paid for their training and work hours.

*Simulation Procedure.* YAPM were prepped more than one week ahead of the simulation encounter that they would be completing one face-to-face and one phone encounter with a standardized mentee. On the day of the simulation practice, YAPM were given 10 minutes to read the door note/case vignette and prepare for the interaction. YAPM were given the opportunity to ask questions to the lead author prior to the entering the simulation encounter. They were able to make notes that they could take into the session with them. YAPM then entered the room with the standardized mentee actor and engaged using interpersonal skills. Encounters had a maximum time of 20 minutes; YAPM could leave the encounter before 20 minutes expired. YAPM were alerted by a knock on the door when there were five minutes remaining in the encounter. In all simulations, YAPM worked with a different standardized mentee actor; they never met with the same actor twice.

Training Materials

For each of the four vignettes, several materials were created. The *standardized mentee guide*, which was the primary training document for the standardized mentee actors, included a case synopsis of the presenting concern, questions for the actor to answer to personalize the backstory, some guidelines for how to respond to YAPM questions and responses, key content related to the vignette (e.g., SMART goals, problem solving steps) which oriented the actor to likely interventions the YAPM may use. A less detailed synopsis (i.e., *door note*) for each vignette was created for the YAPM to read prior to the simulation. The door note contained key background information about the mentee, presenting concern, tasks to complete during the simulation, and a suggestion for how to start the encounter. A *debrief encounter form* as well as two evaluation forms (YAPM self-reflection, Mentee Evaluation) were created to structure the feedback session after each vignette.

The remainder of the appendix includes the guides and forms used during simulation, in the following order:

Vignette A: Ambivalence in Goal Setting. Standardized Mentee Guide

Vignette A: Ambivalence in Goal Setting. Door Note

Vignette B: Ambivalence Toward Group. Standardized Mentee Guide

Vignette B: Ambivalence Toward Group. Door Note

Vignette C: Difficulty with Describing Diagnosis. Standardized Mentee Guide

Vignette C: Difficulty with Describing Diagnosis. Door Note

Vignette D: Difficulty with Locating an Adult Provider. Standardized Mentee Guide

Vignette D: Difficulty with Locating an Adult Provider. Door Note

YAPM Self-Reflection Form

Standardized Mentee Evaluation Form

Debrief Discussion Form (used for all vignettes)

**Standardized Mentee Guide: Scenario A: Ambivalence in goal setting**

**Topic:** Goal Setting

**Anticipated Length:** 20 minutes

**Setting:** In-Person (breakout from group session activity and you are approached after group to check in individually)

**Materials:** Goal setting worksheet

**Case Synopsis:** You know that you are participating in a program about transitioning from pediatric care to adult care. Your caregiver encouraged you to attend so you could learn some tips on how to transition successfully. During the first group session, everyone breaks into small groups with their mentor to work on the goal setting worksheet. You really aren’t sure what goal(s) to set. You start to feel frustrated and feel like this is just a waste of your time.

**Your Name:**

**Age:**

**Medical history:**

Please write some responses to these questions prior to the training:

**If you didn’t have to come to this group, how else would you be spending your time?**

**What three tips specifically did your mother hope that you learned in this group?**

**Which one of those tips do you agree with?**

**Even though your mother encouraged you (ok, just flat out signed you up with no choice!), you do have some concerns about this transition that you wouldn’t mind sorting out. What two concerns about transition do you have?**

**Why are these concerns important to you (desire)?**

**How would you feel or what would be different if you were to solve those concerns (reasons)?**

**What is one small step you have already done to help with your transition (ability, taking steps)?**

**Why would now be a good time to resolve these concerns (and participate in this group) (need)?**

**You’ve set goals in the past (that you see now were unrealistic) and you feel like you failed at them. What is one ineffective goal you set in the past?**

**If someone asked, what more specific or do-able goal would you be open to considering, given your concerns for your health and transition?**

Key Notes

During the encounter

- Don’t disclose everything right at the start. Especially given that you may be feeling frustrated, what might be initially on your mind will be more negative thoughts about your situation. Slowly add in positive aspects about making a change or setting a goal. If they begin to ask you about your reasons or benefits of making a goal/changing your health – really go with it and give them longer/fuller answers. Let your mood be brighter and thoughtful about this side of the conversation. If they ask you for your barriers, (why setting a goal is not what you want to do), you can also give longer responses (although we are hoping they don’t spend a lot of time asking about things that keep you talking about not setting a goal).
- If they start to give you advice (and especially if they haven’t asked if that would be okay or asked for your ideas first), don’t be too interested in the advice. You might acknowledge that it is good, but find a way that it also might not work for you.
- If they ask many closed ended questions (those that you could completely answer with a yes/no or one word answer), just give the short answer. 1-2 of these questions are ok, but we are hoping for them to ask more open-ended questions (“How come…” “what are your thoughts about…”)
- If they try to understand your feelings or your perspective, really let them know what resonates with you and your appreciation for their listening. Let them know when they do understand (“exactly!” “yes”)
- Overall, be generally friendly (even if you are feeling frustrated) – you don’t have to be close and buddies with them, but also stay away from being too “difficult” or argumentative. Ultimately, you are approaching this situation truly feeling uncertain about what you should do.

What the mentor will know

- You seemed frustrated in group
- You struggled to make a goal
- They are going to ask what got in the way.
- Your name, age, and medical diagnosis

What they will not know:

- Why you feel frustrated
- Your past experiences with goals or changes.

**Door Note for Mentor : Scenario A**

**Setting:** In-Person (breakout from group session)

**Anticipated Length:** 20 minutes

**Materials:** Goal setting worksheet

**Case Synopsis:** During the first group intervention session, everyone breaks into pairs with their mentor to work on goal setting. You sit down with your mentee to help her work through the goal setting worksheet and notice her becoming increasingly frustrated with the activity and having a difficult time creating a goal. After the group is complete, you want to be able to speak with her privately and inquire about what might be tough.

**Mentee name:**

**Mentee age:**

**Mentee medical history:**

Your task is to

1. Inquire about her frustration.
2. Be listening and responding to change talk.
3. Be thoughtful of what stage of change she is in.
4. See if you can help her set a goal that is related to her health and something she is willing to do.

You may start the conversation anyway you wish. If it is helpful to have a starting line to get the conversation going, we suggest:

“Hey [name], thanks for taking a few minutes to talk with me after group. I noticed that activity on goal setting seemed frustrating.”

**Standardized Mentee Guide: Scenario B: Ambivalence toward group**

**Topic:** Wanting to Leave the Program

**Anticipated Length:** 20 minutes

**Setting:** Phone (check-in between group sessions)

**Case Synopsis:** You finished the first group session of the peer mentor program last Saturday. You think it went okay but you don’t know if you want to go back. Your mentor calls to check-in with you and you share with him/her your thoughts on whether or not you want to attend the next session.

**Name:**

**Age:**

**Medical history:**

Please write some responses to these questions prior to the training:

**What are three things you liked about group or found helpful?**

**What are two things you didn’t like about group that is leading you to not want to come back?**

**What is your biggest fear about continuing in the group?**

**You did have your own personal reasons for being a part of this group. When it was advertised to you, you thought it was a really good idea, as you have been struggling with certain aspects of your health. What have you been struggling with most recently that you were hopeful group could address (desire/reasons)?**

**What was one other reason that you signed up for this group (reason)?**

**Why did now feel like a good time to come to group (need)?**

**What are some accomplishments you have already had in taking care of your health (loose ability)?**

**If asked, what suggestions would you have to give group another try or to feel better about attending group?**

Key Notes

During the encounter

- Don’t disclose everything right at the start. Especially given that you may be feeling frustrated, what might be initially on your mind will be more negative thoughts about your situation. Slowly add in positive aspects about making a change or sticking with the group. If they begin to ask you about your reasons to want to stay or your opinion about the positive aspects of the group – really go with it and give them longer/fuller answers. Let your mood be brighter and thoughtful about this side of the conversation. If they ask you for your barriers, (why you don’t want to stay), you can also give longer responses (although we are hoping they don’t spend a lot of time asking about things that keep you talking about not coming to group).
- If they start to give you advice (and especially if they haven’t asked if that would be okay or asked for your ideas first), don’t be too interested in the advice. You might acknowledge that it is good, but find a way that it also might not work for you.
- If they ask many closed ended questions (those that you could completely answer with a yes/no or one word answer), just give the short answer. 1-2 of these questions are ok, but we are hoping for them to ask more open-ended questions (“How come…” “what are your thoughts about…”)
- If they try to understand your feelings or your perspective, really let them know what resonates with you and your appreciation for their listening. Let them know when they do understand (“exactly!” “yes”)
- Overall, be generally friendly (even if you are feeling frustrated) – you don’t have to be close and friendly to them, but also stay away from being too “difficult” or argumentative. Ultimately, you are approaching this situation truly feeling uncertain about what you should do.

What the mentor will know

- This is a standard phone check in.
- They are going to ask about your thoughts on the group.
- Your name, age, and medical diagnosis

What they will not know:

- You feel frustrated.
- You are thinking about not coming back to the group.

**Door Note for Mentor : Scenario B**

**Setting:** Phone (check-in between group sessions)

**Anticipated Length:** 20 minutes

**Case Synopsis: T**he first group session of the peer mentor program took place last Saturday. You are calling one of your mentees to check-in with her and see how it went. You noticed that there were times when she seemed checked out and you are wondering how much she felt the group was helpful.

**Mentee name:**

**Mentee age:**

**Mentee medical history:**

Your task is to

1. Inquire about her group experience.
2. Be listening and responding to change talk.
3. Be thoughtful of what stage of change she is in.

You may start the conversation anyway you wish. If it is helpful to have a starting line to get the conversation going, we suggest:

“[ring ring] Hi [mentee name], this is [your name]. As part of our program I will call weekly to check in. How was your experience in group this past weekend?”

**Standardized Mentee Guide: Scenario C: Difficulty with describing diagnosis**

**Topic:** Explaining Your Diagnosis to Someone New

**Related Reading:** [provided mentees with the group intervention materials for this session. May need to provide mentees with some information about guidelines for sharing diagnosis and ways to describe it to others]

**Anticipated Length:** 20 minutes

**Setting:** In-Person (breakout from group session)

**Case Synopsis:** During a group session, everyone breaks into small groups with their mentor to brainstorm how you might explain your diagnosis to someone new. You and your mentor are supposed to discuss some situations when you might explain your diagnosis to someone and how you would do so. You struggle to explain the details of your diagnosis, especially given a time in your past when this didn’t go so well, but you are open to benefits that may come from feeling more at ease with telling people and some positive health outcomes that could come from sharing. Ideally, you would start off as somewhat engaged but uncertain. As the mentor tries to work through the activity, you find yourself feeling slightly more frustrated and you may say that directly to your mentor.

**Name:**

**Age:**

**Medical diagnosis:**

Please write some responses to these questions prior to the training:

**List 3 situations when you might (need to) explain your diagnosis to someone.**

**What would be the key points you would want people to know about your diagnosis?**

**What do most people not understand about your diagnosis?**

**Describe one person you have had to tell in the past where the conversation went well.**

**Describe a time that was uncomfortable when sharing (or you felt forced to share) health information – a time when sharing didn’t go so well (As referenced above in the initial paragraph). What happened? What would you have wished would have been different?**

**What is one barrier you feel like you need to overcome to make telling others (that you trust) about your diagnosis?**

**List 3 benefits of people knowing your diagnosis or health information (maybe friends, extended family that don’t know, teachers, employer).**

**Also, sometimes you feel nervous telling others who need to know (e.g., your nurse or doctor), you acknowledge that this practice in group will help with talking to your doctor/nurse about issues.**

Key Notes

During the encounter

- Don’t disclose everything right at the start. Especially given that you may be feeling uncertain, what might be initially on your mind will be more negative thoughts about sharing. Slowly add in positive aspects about sharing your diagnosis. If they begin to ask you about your reasons to want to share or your opinion about the positive aspects – really go with it and give them longer/fuller answers. Let your mood be thoughtful about this side of the conversation. It is okay to share your past experiences where self-disclosure didn’t go well. Do try to keep the story concise/focused. While there are likely some emotions behind the experience, let your emotions stay in check while describing (you are maybe a 4, 5, 6 of an uncomfortable feeling on a 10-point scale); you are able to distance yourself from the immediate feelings of the situation when it first happened.
- If they start to give you advice (and especially if they haven’t asked if that would be okay or asked for your ideas first), don’t be too interested in the advice. You might acknowledge that it is good, but find a way that it also might not work for you.
- If they ask many closed ended questions (those that you could completely answer with a yes/no or one word answer), just give the short answer. 1-2 of these questions are ok, but we are hoping for them to ask more open-ended questions (“How come…” “what are your thoughts about…”)
- If they try to understand your feelings or your perspective, really let them know what resonates with you and your appreciation for their listening. Let them know when they do understand (“exactly!” “yes”)
- Overall, be generally friendly (even if you are feeling uncertain) – you don’t have to be close and friendly to them, but also stay away from being too “difficult” or argumentative. Ultimately, you are approaching this situation truly feeling uncertain about what you should do.

What the mentor will know

- This is a one-on-one time with you during a group session.
- They are going to ask about how you may share your diagnosis with others.
- Your name, age, and medical diagnosis

What they will not know:

- You feel uncertain about sharing your diagnosis with others.
- You had a tough experience with sharing in the past.

**Door Note for Mentor : Scenario C**

**Setting:** In-Person (breakout from group session)

**Anticipated Length:** 20 minutes

**Case Synopsis:** During a group intervention session, everyone breaks into small groups with their mentor to brainstorm how you might explain your diagnosis to someone new (Session 1a). You and your mentee are supposed to discuss some situations when she might explain her diagnosis to someone and how she would do so.

**Mentee name:**

**Mentee age:**

**Mentee medical history:**

Your task is to

1. Be listening and responding to change talk.
2. Be thoughtful of what stage of change she is in.
3. To the best of your ability, consider open-ended questions, affirmations, and reflections as tools to get to know your mentee better and address any concerns.
4. If you give advice, ask permission first.

You may start the conversation anyway you wish. If it is helpful to have a starting line to get the conversation going, we suggest:

*“As we start on this assignment, what would be some things you would want others to know about your diagnosis?”*

**Standardized Mentee Guide: Scenario D: Difficulty with locating adult provider**

**Topic:** Didn’t Research an Adult Provider

**Anticipated Length:** 20 minutes

**Setting:** Phone (check-in between group sessions)

**Case Synopsis:** You have finished two group sessions of the peer mentor program. Your mentor calls to check in with you and asks if you have selected an adult provider to research and evaluate as a potential provider (which was one of your homework assignments). You haven’t done this assignment yet. If your mentor begins the conversation with getting to know you a little better and asking about your week, please engage with them about this. If they don’t do this, it is okay (you don’t feel upset by them not checking in).

Homework assignment assigned: *Select an adult provider and evaluate for characteristics/criteria. You can call or email them and should have a list of questions ready for them or the secretary. Map how to get there and how to park.*

**Name:**

**Age:**

**Medical history:**

Please write some responses to these questions prior to the training:

**What has gotten in the way of this assignment (consider one barrier in addition to time constraints)?**

**Which barrier are you most open to problem solving?**

**What are some possible solutions to this barrier from your perspective?**

**Did you understand that assignment completely or still have questions? If, you have questions what are they?**

**What are 3 reasons why this assignment is a good idea?**

**What are 5+ qualities you are looking for in an adult provider?**

**What makes now an important time to find a new adult provider?**

**What is one frustration you have with your current pediatric provider or their office? How would this be better in an adult office?**

**In the past, you had to choose a new dentist because your dental insurance didn’t cover your former provider. Your mom helped some with this, but didn’t do all the work for you. What 2 steps did you do to successfully get to a new dentist? How might those steps be applicable to finding a new doctor?**

**What questions had you already thought might be good to ask a new provider? List 3.**

Key Notes

During the encounter

- Don’t disclose everything right at the start. Be open/honest; you can immediately share that you did not do the assignment, but you do not have to share all the reasons why up front. Slowly add in details as they come up in open-ended questions or with reflections. If they begin to ask you about your reasons to find an adult provider – do try to identify several details. Let your mood be ambivalent and easy to lean toward finding the provider if you can figure out the next step. Attempt to have barriers that are resolvable in a conversation; the barrier to the adult provider does not have to be completely resolved, but consider a next, do-able step that is in your control
- If they start to give you advice (and especially if they haven’t asked if that would be okay or asked for your ideas first), don’t be too interested in the advice. You might acknowledge that it is good, but find a way that it also might not work for you.
- If they ask many closed ended questions (those that you could completely answer with a yes/no or one word answer), just give the short answer. 1-2 of these questions are ok, but we are hoping for them to ask more open-ended questions (“How come…” “what are your thoughts about…”)
- If they try to understand your feelings or your perspective, really let them know what resonates with you and your appreciation for their listening. Let them know when they do understand (“exactly!” “yes”)
- Overall, be generally friendly (even if you are feeling uncertain) – you don’t have to be close and friendly to them, but also stay away from being too “difficult” or argumentative. Ultimately, you are approaching this situation truly feeling uncertain about what you should do.

What the mentor will know

- This is a typical phone check-in where they will check in on how things are going generally and the assignments from group.
- Your name, age, and medical diagnosis

What they will not know:

- You have not completed the assignment.
- Your reasons / situations for feeling stuck in this assignment.

**Door Note for Mentor : Scenario D**

**Setting:** Phone (check-in between group sessions)

**Anticipated Length:** 20 minutes

**Case Synopsis:** Your mentee has finished the first two group sessions of the peer mentor program. You call to check in with your mentee and to ask if she has selected an adult provider to research and evaluate as a potential provider (which was one of the homework assignments).

Homework assignment: *Select an adult provider and evaluate for characteristics/criteria. You can call or email them and should have a list of questions ready for them or the secretary. Map how to get there and how to park.*

**Mentee name:**

**Mentee age:**

**Mentee medical history:**

Your task is to

1. Follow up on the homework assignment.
2. Continue to develop rapport.
3. Be listening and responding to change talk.
4. Be thoughtful of what stage of change she is in.
5. To the best of your ability, consider open-ended questions, affirmations, and reflections as tools to get to know your mentee better and address any concerns.
6. If you give advice, ask permission first.

You may start the conversation anyway you wish. If it is helpful to have a starting line to get the conversation going, we suggest:

*“Hi, this is [name], your Mentor from the young adult group last Saturday. I just wanted to call and check in with you this week. OR*

*“Hi, this is [name], your Mentor from the young adult group last Saturday. I just wanted to call to see how the between-group activities have been going. Last week we talked about taking the first steps to finding an adult provider – how is that going?”*

**YAPM Self-reflection**

Please respond to the following questions.

1. (circle one) At what stage was your mentee at the beginning of your conversation?
   1. Pre-contemplation
   2. Contemplation
   3. Preparation
   4. Action
   5. Maintenance
   6. Relapse
2. (circle one) At what stage was your mentee at the end of your conversation?
   1. Pre-contemplation
   2. Contemplation
   3. Preparation
   4. Action
   5. Maintenance
   6. Relapse
3. Which examples of change talk did you hear?
   1. Desire:
   2. Ability:
   3. Reasons:
   4. Need:
   5. Commitment/Activation:
   6. Taking steps:
4. What other statements of change were you hoping to hear from your mentee? (What do you wished they would have said?)
5. (circle) How strong was your righting reflex (your desire to give advice)?
   1. Very strong
   2. Strong
   3. Moderate
   4. Mild
   5. Not present
   6. What advice did you give?
6. Give an example of a time when you didn’t follow-up with a question (e.g., reflection, advice, affirmation, compliment)
   1. How did it go?
   2. What was your mentee’s response?
7. What was one positive non-verbal aspect of your communication?
8. What is one skill/goal you would like to work on as a mentor after participating in this standardized mentee experience?
9. On a scale of 1-10 with 1=not at all well and 10=extremely well, how well would you rank your interaction as a mentor?

**Standardized Mentee Evaluation**

Please respond to the following questions.

1. This mentor’s non-verbal language (eye contact, relaxed body, open posture) was excellent.
   1. strongly agree
   2. agree
   3. slightly agree
   4. slightly disagree
   5. disagree
   6. strongly disagree
2. This mentor used words, phrases, and questions that I easily understood.
   1. strongly agree
   2. agree
   3. slightly agree
   4. slightly disagree
   5. disagree
   6. strongly disagree
3. This mentor took time to understand my side/position and personal reasons for my actions.
   1. strongly agree
   2. agree
   3. slightly agree
   4. slightly disagree
   5. disagree
   6. strongly disagree
4. This mentor took my opinion seriously.
   1. strongly agree
   2. agree
   3. slightly agree
   4. slightly disagree
   5. disagree
   6. strongly disagree
5. This mentor allowed me to think of my own solutions to my problem before giving me his or her opinion or advice.
   1. strongly agree
   2. agree
   3. slightly agree
   4. slightly disagree
   5. disagree
   6. strongly disagree
6. I felt comfortable expressing myself with this mentor.
   1. strongly agree
   2. agree
   3. slightly agree
   4. slightly disagree
   5. disagree
   6. strongly disagree
7. I found myself talking more about change as opposed to barriers or problems with changing
   1. strongly agree
   2. agree
   3. slightly agree
   4. slightly disagree
   5. disagree
   6. strongly disagree
8. Of the pairs listed below, circle the one that relates more to your experience as a mentee with this mentor:
9. Partnership VS Expert
10. Accepting of you VS judgmental of you
11. Compassionate toward you VS not compassionate toward you
12. Asking your ideas and opinions more often VS giving you his or her opinions more often
13. Overall, what is one thing this person did well as a mentor?
14. Overall, what is one goal you would suggest for this mentor / what is one thing you would ask them to improve?
15. Yes or No? Did you feel at the end of this encounter that your opinion changed (e.g., as a mentee would you have acted on your SMART goal or been more likely to continue in group than when the encounter started?

**Debriefing with the Mentor**

Prompts led by the Trainer to discuss following each simulation.

What do you feel like you did well in this encounter?

What is one area you would like to improve on? Or one aspect of this encounter that you would do different?

What felt natural during this conversation with your mentee?

What felt awkward or uncomfortable? Where was a place you felt you were “stuck?”

*Would you mind if we share a few observations?* (modeling MI permission seeking)

2-3 Areas of Strength:

1 Area of Improvement:

Collaborative goal for continued practice:
